# A Rare Case of Primary Sjogren’s Syndrome Coexisting With Gilbert Syndrome

**DOI:** 10.7759/cureus.45521

**Published:** 2023-09-19

**Authors:** Lin Zhang, Zhichun Liu, Leixi Xue

**Affiliations:** 1 Rheumatology and Immunology, The Second Affiliated Hospital of Soochow University, Suzhou, CHN

**Keywords:** osteoporosis, unconjugated bilirubin, hyperbilirubinemia, primary sjogren's syndrome, gilbert syndrome

## Abstract

Gilbert syndrome (GS) is an autosomal recessive inherited bilirubin metabolism disorder characterized by chronic unconjugated hyperbilirubinemia in the absence of hemolysis and liver disease. Primary Sjogren's syndrome (pSS), mainly occurring in women, is a common connective tissue disease (CTD) wherein bilirubin levels are generally reduced. We report a rare case of pSS coexisting with GS. A 35-year-old female patient presented to our hospital with pSS and chronic unconjugated hyperbilirubinemia, for which low-dose methylprednisolone was ineffective. The patient’s liver function test results were normal, serological tests for hepatitis virus were negative, and abdominal ultrasound did not indicate abnormal liver morphology. Bone mineral density determination showed that the Z scores of the left femoral neck and lumbar spine were -1.9 and -2.6, respectively, with T scores of -2.1 and -2.8, respectively. Full-exon sequencing revealed a homozygous TA insertion in the TATA box (A(TA)7TAA) and a heterozygous base substitution from C to A at nucleotide position 686 in exon 1 (c.686C>A) in the uridine glucuronosyltransferase 1A1 (UGT1A1) gene. Therefore, the patient was diagnosed with pSS, GS, and osteoporosis. The dose of methylprednisolone was then reduced and gradually stopped, and treatment for osteoporosis was strengthened. To our knowledge, this is the first report of pSS with GS. It is important to clarify the cause of hyperbilirubinemia in patients with CTD, including pSS, which affects the formulation of correct treatment plans.

## Introduction

Gilbert syndrome (GS) is a benign bilirubin metabolic disorder characterized by chronic unconjugated hyperbilirubinemia in the absence of hemolysis and liver disease. It was first reported by Gilbert and Lereboulet in 1901 [1]. Gilbert syndrome occurs in 5% to 10% of the global population, with a male-to-female ratio of 1.5 to 10:1 [2,3]. Under physiological conditions, unconjugated bilirubin combines with glucuronic acid to form conjugated bilirubin via uridine glucuronosyltransferase 1A1 (UGT1A1) in the liver [1]. Mutations in the UGT1A1 gene can result in decreased enzyme activity, leading to increased serum unconjugated bilirubin levels [4]. Therefore, UGT1A1 mutations are considered to be the pathological basis of GS. Common polymorphic mutations in the UGT1A1 gene in GS include a TA insertion mutation in the TATA box and a missense mutation in one of the exons of the gene [5]. The substitution of the A(TA)7TAA sequence for the normal A(TA)6TAA (UGT1A1*28) and the insertion of additional TA sequences diminish the affinity of the binding protein to the TATA box, resulting in the downregulation of gene expression, thereby reducing the activity of the UGT1A1 enzyme [6]. Similarly, c.211G>A in exon 1 (UGT1A1*6) is a common missense mutation that causes the 71st amino acid of UGT1A1 to change from glycine to arginine, thereby affecting enzyme activity [7]. Mutations in other alleles, such as c.686C>A in exon 1 (UGT1A1 * 27) and c.1091C>T in exon 4 (UGT1A1 * 63), have also been confirmed to cause unconjugated hyperbilirubinemia [5].

Primary Sjogren's syndrome (pSS) is a common connective tissue disease (CTD) that more frequently affects women, with a global incidence ranging from 0.05% to 0.5%. It mainly involves the exocrine glands, resulting in dry mouth, dry eyes, and caries. It can lead to liver damage, causing an elevation in serum alanine aminotransferase (ALT) and aspartate aminotransferase (AST) levels [8]. Primary Sjogren's syndrome can also overlap with autoimmune hepatitis or primary biliary cholangitis (PBC), showing an increase in serum AST, ALT, alkaline phosphatase (ALP), and gamma-glutamyl transferase (GGT) levels. However, patients with pSS rarely exhibit elevated bilirubin levels. In contrast, a study found that serum total bilirubin, unconjugated bilirubin, and conjugated bilirubin levels in patients with pSS were significantly reduced [9]. In this study, we report a case of pSS with hyperbilirubinemia, wherein the patient was finally diagnosed with concurrent GS.

## Case presentation

The patient was a 35-year-old female with a two-year history of pSS. She was treated with prednisone (5 mg/day), hydroxychloroquine (400 mg/day), total glucosides of paeony (600 mg/day), alfacalcidol (0.5 μg/day), and calcium carbonate (600 mg/day). The patient had regular menstruation and an unremarkable family history. In January 2022, serum total bilirubin and unconjugated bilirubin were noted to be elevated. However, the serum levels of ALT, AST, ALP, GGT, and bile acid were normal (Table 1), and she had no itching, jaundice, or other subjective complaints. In July 2022, prednisone was replaced with methylprednisolone (6 mg/day), and ursodeoxycholic acid (UDCA; 250 mg/day) was added. Nonetheless, bilirubin levels did not change significantly after one month of the new regimen (Table 1).

**Table 1:** Liver function test results of the patient

Liver function index	2022/01/19	2022/02/17	2022/04/10	2022/07/10	2022/08/08	2022/08/13	Normal range
Total bilirubin (μmol/L)	51.7	37.8	39.1	54.4	60.2	50.9	3.4–23.0
Unconjugated bilirubin (μmol/L)	43.8	24.4	32.9	47.4	43.1	36.7	0.0–15.0
Conjugated bilirubin (μmol/L)	7.9	13.4	6.2	7.0	17.1	14.2	0.0–4.0
Alanine aminotransferase (U/L)	20	16	15	13	8.4	10	7–40
Aspartate aminotransferase (U/L)	22	19	18	18	16.4	16	13–35
Alkaline phosphatase (U/L)	46	47	43	38	37	39	35–100
Gamma-glutamyl transferase (U/L)	13	13	13	12	9	8	7–45
Bile acid (μmol/L)	2.10	6.10	3.50	1.10	4.6	6.38	0.14–9.66

**Figure 1:**
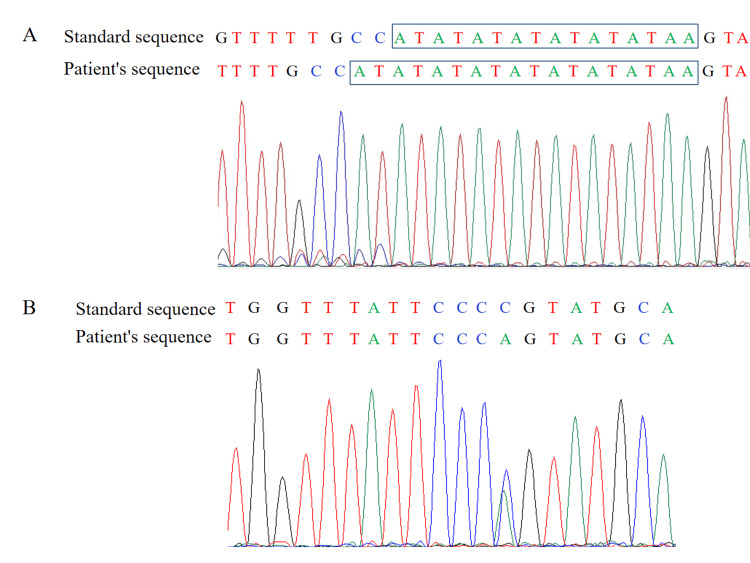
Full-exon sequencing revealed two mutations in the UGT1A1 gene of the patient A: Homozygous TA insertion in the TATA box (A(TA)7TAA; B: Heterozygous base substitution from C to A at nucleotide position 686 in exon 1 (c.686C>A) UGT1A1: Uridine glucuronosyltransferase 1A1

On August 12, 2022, she was referred to the Department of Rheumatology and Immunology for chronic hyperbilirubinemia and anxiety. The liver function tests still showed hyperbilirubinemia, characterized by elevated unconjugated bilirubin (Table 1). Serology tests for the hepatitis virus were negative, and an abdominal ultrasound did not indicate abnormal liver morphology. The immunological test results were as follows: antinuclear antibody (ANA), positive, nuclear membrane type 1:1000; anti-Sjögren's-syndrome-related antigen A autoantibodies (SSA)/Ro60 antibody, positive; anti-ribosomal P protein antibody, positive; antinuclear ribonucleoprotein antibody/anti-Smith antibody, positive; anti-SP100 antibody, positive; anti-GP210 antibody, positive; immunoglobulin G, 15.80 g/L (7.51-15.60 g/L); complement 3 (C3), 0.58 g/L (0.79-1.52 g/L); C4, 0.129 g/L (0.160-0.380 g/L). Serum beta C-terminal cross-linked telopeptides of type Ⅰ collagen (β-CTx) level was 690 pg/mL (100-650 pg/mL), 25-hydroxyvitamin D level was 15.64 ng/mL (≥ 30 ng/mL), and the levels of N-terminal propeptides of type I procollagen, osteocalcin, and parathyroid hormone were all normal. Bone mineral density determination was obtained with central dual-energy X-ray absorptiometry. The Z scores of the left femoral neck and lumbar spine were -1.9 and -2.6, respectively, and the T scores were -2.1 and -2.8, respectively. With the consent of the patient, full-exon sequencing was carried out, and the results revealed a homozygous TA insertion in the TATA box (A(TA)7TAA) and a heterozygous base substitution from C to A at nucleotide position 686 in exon 1 (c.686C>A) in the UGT1A1 gene (Figure 1). Therefore, the patient was diagnosed with pSS, GS, and osteoporosis.

We educated the patient regarding the characteristics of GS, a benign disease that generally does not require special treatment, and the factors that may lead to hyperbilirubinemia, such as fasting, physical exercise, diarrhea, emotional tension, infection, pregnancy, and abnormal menstruation. The patient’s anxiety subsequently improved. The patient’s treatment plan was also revised to address osteoporosis. The dose of methylprednisolone was reduced to 4 mg/day and then slowly tapered. Alendronate sodium (70 mg/week) was added to her regimen, and the doses of alfacalcidol and calcium carbonate were increased to 1.0 μg/day and 1,200 mg/day, respectively. The patient was recommended to continue UDCA treatment at an increased dose of 250 mg twice daily, as UDCA potentially mitigates the harmful effects of hyperbilirubinemia on the bone.

## Discussion

The present study reported the case of a patient with pSS and chronic hyperbilirubinemia characterized by elevated levels of unconjugated bilirubin. A homozygous TA insertion in the TATA box (A(TA)7TAA) and a heterozygous base substitution (c.686C>A) in exon 1 were identified by full-exon sequencing, establishing the diagnosis of GS. Moreover, the results of dual-energy X-ray absorptiometry indicated decreased T and Z scores, meeting the diagnostic standard for osteoporosis. Although the patient developed anti-SP100 and anti-GP210 antibodies specific for PBC, repeated liver function results showed normal ALP, GGT, and bile acid levels. Thus, there was not enough evidence to support the diagnosis of PBC.

Gilbert syndrome is considered an autosomal recessive bilirubin metabolism disorder, but some studies believe it is an autosomal dominant disease with incomplete penetrance [1,10]. The decrease in enzyme activity caused by the UGT1A1 gene mutations is considered the pathological basis of GS. A homozygous A(TA)7TAA (UGT1A1*28) mutation was the most commonly found genetic alteration in Caucasian and African populations [3,11]. However, in Chinese individuals, the most common mutation in GS was 3279T>G, a base substitution in the phenobarbital response-enhancing motif, accounting for 36.3% of mutations, followed by A(TA)7TAA (30.6%) [12]. The present patient not only had a homozygous A(TA)7TAA sequence in the TATA box but also had a heterozygous c.686C>A mutation in exon 1, which resulted in a substitution of glutamine for proline. The combination of this mutation is consistent with that of a patient in Japan who presented with the lowest serum bilirubin concentration among the seven patients reported [13].

Gilbert syndrome is characterized by intermittent hyperbilirubinemia, particularly unconjugated hyperbilirubinemia, despite normal physiological liver function and a lack of inflammation or fibrosis. The level of serum total bilirubin fluctuates from 29 to 100 μmol/L [14]. Therefore, the clinical symptoms of patients with GS are mild and mainly manifest as mild and fluctuating jaundice [3]. Although unconjugated bilirubin can be neurotoxic at very high concentrations (> 300 μmol/L), the elevated concentrations of total bilirubin in GS are not sufficient to cause neurological symptoms [1]. Therefore, GS does not require specialized treatment, and the clinical focus is to establish a diagnosis and distinguish it from other hepatic diseases. Once diagnosed, medication and genetic counseling may be provided to address the condition and eliminate patients’ tension and anxiety. As a hepatic enzyme inducer, phenobarbital promotes the metabolism of unconjugated bilirubin, causing jaundice to subside, and may be tried in anxious patients. 

Osteoporosis was also diagnosed in the patient. Osteoporosis may have serious adverse effects on young women’s lives and work in the future, making active treatment particularly important. Therefore, the patient was prescribed alendronate sodium, and the dose of methylprednisolone was reduced. In addition to the use of glucocorticoids and reduced serum levels of 25-hydroxyvitamin D, hyperbilirubinemia may be another important factor that contributed to the patient’s osteoporosis. Unconjugated bilirubin seen in the serum of jaundiced patients has been shown to result in dose-dependent decreases in osteoblast viability and increases in osteoblast defects and apoptosis [15,16]. Further research also showed that bilirubin upregulates apoptotic genes and colony-stimulating factor 2 while downregulating anti-apoptotic genes in osteoblasts [17]. In contrast, bilirubin increased the viability and decreased the apoptosis of osteoclasts [18]. The increased serum β-CTx level in the present patient may indicate an enhancement of osteoclast activity. These findings support the deleterious consequences of hyperbilirubinemia, resulting in disturbed bone formation and increased bone absorption related to osteoporosis.

Bisphosphonates, calcium supplements, and vitamin D supplements effectively increase bone mass in patients with cholestatic liver disease and hyperbilirubinemia [19]. However, there is currently no specific treatment to counter the harmful effect of increased bilirubin. Ursodeoxycholic acid, an endogenous hydrophilic bile acid used for the treatment of chronic cholestasis, has been shown to increase osteoblast differentiation and mineralization, reduce osteoblast apoptosis, and neutralize the detrimental effects of bilirubin and sera from jaundiced patients on osteoblasts [16]. It also inhibits the effect of bilirubin on osteoclasts [18]. Therefore, UDCA may be favorable for the bones of patients with hyperbilirubinemia, making it a good option for the present patient. Thus, its dosage was increased to 250 mg twice daily.

This case emphasizes the importance of clarifying the cause of hyperbilirubinemia in patients with CTD, including pSS. First, it can avoid overtreatment and prevent unnecessary glucocorticoid use or dosage increases. Second, identifying the cause of hyperbilirubinemia can alleviate anxiety and tension, which is conducive to patients’ mental health. Third, for patients with chronic hyperbilirubinemia, it is necessary to regularly conduct bone mineral density screening and actively prevent the occurrence of osteoporosis to decrease the risk of pathological fractures.

The incidence of GS is as high as 5% to 10%, and CTDs are also common autoimmune diseases. However, only one study of systemic lupus erythematosus with GS has been retrieved [2], and no other case of CTD, including pSS, coexisting with GS has been reported. As the final product of hemoglobin metabolism, serum bilirubin, at low concentrations, functions as a strong endogenous antioxidant and plays an anti-inflammatory and immunosuppressive role, protecting against many diseases. Gilbert syndrome has been associated with a decreased prevalence of ischemic heart disease, diabetes, microvascular complications, and endometrial cancers [20]. Thus, it is speculated that GS may protect patients from CTD, and the association between GS and CTD warrants further study. However, we cannot ignore that some patients with CTD accompanied by hyperbilirubinemia may have been misdiagnosed. Therefore, GS should be differentiated in clinical practice.

## Conclusions

The present study reported a young female patient with pSS and hyperbilirubinemia who was finally diagnosed with GS. It was also found that the patient suffered from osteoporosis during the screening process. It is important to clarify the cause of hyperbilirubinemia in patients with CTD, including pSS, which affects the formulation of appropriate treatment plans. Gilbert syndrome needs to be considered when chronic unconjugated hyperbilirubinemia is present, which can be diagnosed by full-exon sequencing. In addition, the role of bilirubin in CTD still needs to be further elucidated.
